# iGEMDOCK: a graphical environment of enhancing GEMDOCK using pharmacological interactions and post-screening analysis

**DOI:** 10.1186/1471-2105-12-S1-S33

**Published:** 2011-02-15

**Authors:** Kai-Cheng Hsu, Yen-Fu Chen, Shen-Rong Lin, Jinn-Moon Yang

**Affiliations:** 1 Institute of Bioinformatics and Systems Biology, National Chiao Tung University, Hsinchu, 30050, Taiwan; 2 Department of Biological Science and Technology, National Chiao Tung University, Hsinchu, 30050, Taiwan; 3 Core Facility for Structural Bioinformatics, National Chiao Tung University, Hsinchu, 30050, Taiwan

## Abstract

**Background:**

Pharmacological interactions are useful for understanding ligand binding mechanisms of a therapeutic target. These interactions are often inferred from a set of active compounds that were acquired experimentally. Moreover, most docking programs loosely coupled the stages (binding-site and ligand preparations, virtual screening, and post-screening analysis) of structure-based virtual screening (VS). An integrated VS environment, which provides the friendly interface to seamlessly combine these VS stages and to identify the pharmacological interactions directly from screening compounds, is valuable for drug discovery.

**Results:**

We developed an easy-to-use graphic environment, *i*GEMDOCK, integrating VS stages (from preparations to post-screening analysis). For post-screening analysis, *i*GEMDOCK provides biological insights by deriving the pharmacological interactions from screening compounds without relying on the experimental data of active compounds. The pharmacological interactions represent conserved interacting residues, which often form binding pockets with specific physico-chemical properties, to play the essential functions of a target protein. Our experimental results show that the pharmacological interactions derived by *i*GEMDOCK are often hot spots involving in the biological functions. In addition, *i*GEMDOCK provides the visualizations of the protein-compound interaction profiles and the hierarchical clustering dendrogram of the compounds for post-screening analysis.

**Conclusions:**

We have developed *i*GEMDOCK to facilitate steps from preparations of target proteins and ligand libraries toward post-screening analysis. *i*GEMDOCK is especially useful for post-screening analysis and inferring pharmacological interactions from screening compounds. We believe that *i*GEMDOCK is useful for understanding the ligand binding mechanisms and discovering lead compounds. *i*GEMDOCK is available at http://gemdock.life.nctu.edu.tw/dock/igemdock.php.

## Background

Structure-based drug design is widely used to identify lead compounds with the growing availability of protein structures [1-3]. Many tools (*e.g.*, GEMDOCK [4], DOCK [5], AutoDock [6], and GOLD [7] ) have been developed for virtual screening (VS) and successfully identified lead compounds for some target proteins. However, the accuracy of these docking tools remained intensive because of the incomplete understandings of ligand binding mechanisms [1-3]. In addition, most of scoring functions are lack of pharmacological interactions that are essential for ligand binding or biological functions [8]. Recently, some approaches have been proposed to derive pharmacological interactions from known compounds [8-10]. These approaches apparently increase hit rates for identifying the active compounds which are often similar to the known compounds. In addition, these approaches are often unable to be applied for new targets, which have no known active compounds.

Generally, a VS procedure consists of four main steps: preparations of the target protein and the compound library, docking and post-screening analysis (*e.g.*, clustering compounds and pharmacological interactions). Most docking programs (e.g. DOCK [5] and AutoDock [6]) only provide docked poses or loosely coupled these steps. They often provided limit ability for post-screening analysis. Therefore, a VS framework, providing an easy-to-use graphic and integrated environment, is an emergent task for drug discovery.

To address these issues, we have developed a structure-based VS framework, named *i*GEMDOCK, from preparations through to post-screening analysis. *i*GEMDOCK is an integrated environment, which integrates the heavily modified and enhanced in-house tool GEMDOCK, protein-ligand profiles, pharmacological interactions, and compound clusters. GEMDOCK was comparative to several docking tools (e.g. DOCK [5] and GOLD [7]) and has been successfully applied to identify new inhibitors and new binding sites for some targets [4,8,11-14]. Notably, *i*GEMDOCK derives the pharmacological interactions from screening compounds without using a set of known active compounds. The pharmacological interactions, which often form binding pockets with specific physico-chemical properties of the target protein, can represent conserved interactions between the interacting residues and the screening compounds. We initially validated the pharmacological interactions on three therapeutic protein targets, including estrogen receptor α for antagonists and agonists and thymidine kinase. Our experimental results show that the derived pharmacological interactions are often essential for the ligand binding or maintaining biological functions for these targets. In addition, *i*GEMDOCK provided a post-screening analysis module, which is convenient for clustering compounds and visualizing the pharmacological interactions by interaction profiles. We believe that *i*GEMDOCK is useful for drug discovery and identifying essential residues and interactions for understanding the binding mechanisms.

## Methods

### Preparations of proteins and compound sets

To initially validate the pharmacological interactions, we selected three therapeutic protein targets, including estrogen receptor α for agonists (ERA, PDB code 1gwr [15]), estrogen receptor α for antagonists (ER, PDB code 3ert [16]), and thymidine kinase (TK, PDB code 1kim [17]) because these proteins were well studied. The catalytic mechanisms, biological functions, key functional residues, and active compounds of the three targets were available in the literatures. Estrogen receptor is an important therapeutic target for osteoporosis and breast cancer [18], and TK is a drug target for the therapy of herpes simplex virus type-1 [19]. Moreover, we also evaluate the docking and screening accuracy of *i*GEMDOCK. For docking, a highly diverse dataset comprising 305 protein-compound complexes (*i.e.*, CCDC/Astex set [20]) was selected; for screening, we prepared 10 known active compounds and 990 compounds were randomly selected from Available Chemical Directory (ACD) proposed by Bissantz *et al.*[21] for each therapeutic protein target.

### Main procedure

**Figure 1:**
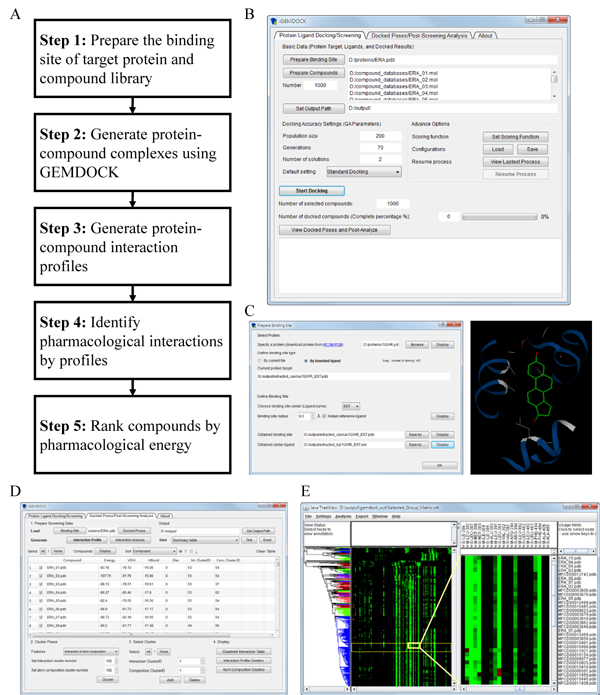
**Overview of iGEMDOCK using estrogen receptor α as the example**. (A) Main steps. (B) The protein-ligand docking/screening interface. This interface provides an easy way for the preparations of binding site and screening compounds, the customization of docking parameters, and monitoring the docking progress. (C) The binding site preparation interface in the docking/screening stage. iGEMDOCK allows users to directly set the binding site and visualize the structure. (D) The post-screening analysis interface displays the protein-ligand complex structures, clusters, and ranks of screening compounds. (E) The hierarchical tree presents the compound similarities using compound structures or protein-compound interactions.

*i*GEMDOCK is an integrated VS environment from preparations through post-screening analysis with pharmacological interactions (Fig. 1A). First, *i*GEMDOCK provides interactive interfaces to prepare both the binding site of the target protein and the screening compound library (Figs. 1B and 1C). Each compound in the library is then docked into the binding site by using the in-house docking tool GEMDOCK [4]. Subsequently, *i*GEMDOCK generates protein-compound interaction profiles of electrostatic (E), hydrogen-bonding (H), and van der Waals (V) interactions. Based on these profiles and compound structures, *i*GEMDOCK infers the pharmacological interactions and clusters the screening compounds for the post-screening analysis (Figs. 1D and 1E). Finally, *i*GEMDOCK ranks and visualizes the screening compounds by combining the pharmacological interactions and energy-based scoring function of GEMDOCK.

### Mining pharmacological interactions

**Figure 2:**
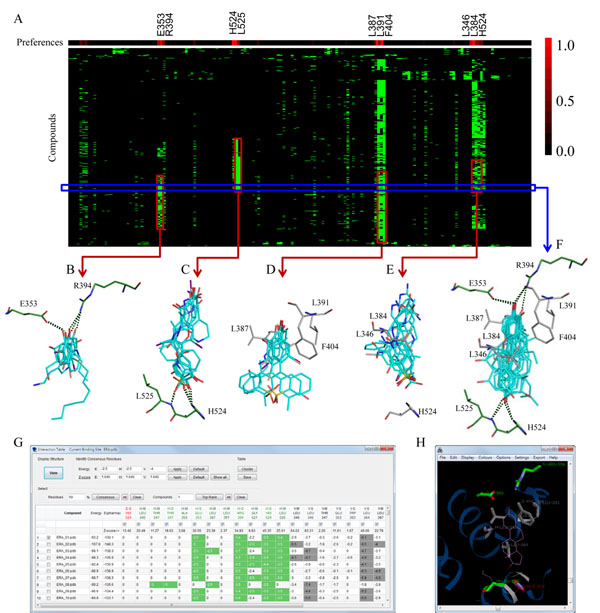
**Interaction profiles and pharmacological interactions**. (A) Protein-compound interaction profiles of ERA. The conserved interacting residues (B) E353 and R394 as well as (C) L525 and H524 form hydrogen bonds with the screening compounds. On average, 70% (>700) and 40% (>400) screening compounds have vdW contacts with (D) the upper hydrophobic pocket residues (L387, L391 and F404) and (E) the bottom hydrophobic pocket residues (L346, L384, and H524), respectively. (F) 10 active compounds highly agree to form hydrogen bonds with residues R394, E353, L525, and H524. (G) The interactions and (H) visualizations of pharmacological interactions in the post-screening analysis interface.

*i*GEMDOCK mines the pharmacological interactions based on protein-compound interaction profiles (Fig. 2). The size of each profile is *N*×*2K* where *N* and *K* are the numbers of screening compounds and interacting residues of the target protein, respectively. Here, an interacting residue is divided into two interacting groups: main and side chains. A profile *P*(*I*) with type *I* (E, H, or V) is given as (Fig. 2A):

where *p_i_*_,_*_j_* is a binary value (0 or 1) for the compound *i* interacting to the residue group *j*. In the E and H profiles, the *p_i_*_,_*_j_* is set to 1 (green) if hydrogen-bonding or electrostatic interactions are yielded between the compound *i* and the residue *j* (energy ≤ -2.5 kcal/mol); otherwise, *p_i_*_,_*_j_*=0 (black). For the V profile, *p_i_*_,_*_j_* = 1 if the interacting energy is less than -4 kcal/mol (Fig. 2A).

After the generations of the profiles, we identified the pharmacological interactions. For each interacting residue group, the *z*-score value is used to measure the interaction conservation between the interacting groups and the screening compounds. For computing the z-scores of interacting groups in a profile, 1000 randomly shuffled profiles are utilized to obtain the standard deviation (*σ*) and mean (*μ*). The *z*-score of the interacting residue group *j* is defined as , where *f_j_* is given as , where *N* is the number of screening compounds. Finally, we normalize the *z*-score value as follows:(1)

where *W_j_* is the interaction conservation of the residue group *j* related to the largest z-score (*z_max_*) among all of interacting groups in the binding site. Here, an interaction conservation is viewed as a pharmacological preference and an interaction is considered as the pharmacological interaction if *W_j_* ≥0.4. For example, for the hydrogen profile of the target ERA, the pharmacological preferences of E353 and R394 are 0.64 and 0.80, respectively; for the V profile, the preferences of L387, L391, and F404 are 1.00, 0.61, and 0.90, respectively (Fig. 2B). In this case, over 300 (>30%) screening compounds form hydrogen bonds with the residues E353 or R394 by polar moieties (*e.g*., hydroxyl group (27%), carboxyl group (20%), sulfuric acid monoester (9%), ketone (8%), and phosphoric acid monoester (6%)). Moreover, the aromatic rings of the screening compounds are often sandwiched by vdW interacting residues L387, L391, and F404 (Fig. 2D).

Based on the pharmacological interactions, we developed a pharmacological scoring function for identifying the active compounds from thousands of screening compounds. The pharmacological scoring function is given as(2)

where *E_GEMDOCK_* is the docked energy of GEMDOCK and *E*(E)*_pharma_*, *E*(H)*_pharma_*, and *E*(V)*_pharma_* are the pharmacological scores of electrostatics, hydrogen-bonding, and vdW interactions, respectively. The *E*(*I*)*_pharma_* with interaction type *I* (i.e., E, H, or V) is defined as

where *e_j_* is the energy obtained by the GEMDOCK scoring function for the residue group *j*. Finally, *i*GEMDOCK provides the ranks of energy-based and pharmacological scoring functions for all screening compounds.

### Implementation of iGEMDOCK

*i*GEMDOCK is an easy-to-use VS environment and includes three main modules (Fig. 1): docking and virtual screening tool (GEMDOCK); post-screening analysis methods; and visualization tools (RasMol [22] and Java Treeview [23]). We employed ERA as an example to present these modules, procedures and features of *i*GEMDOCK.

For protein-ligand docking/screening module, *i*GEMDOCK provides an interactive interface for the preparations of the binding site and compound library; setting docking parameters; and monitoring progress status (Fig. 1B). For most docking tools, users usually need to prepare the binding site structure and compound library through complicated steps (*e.g.*, add hydrogen atoms and generate the grids of the protein). Here, *i*GEMDOCK provides a straightforward method to derive the binding site from the bounded ligand. For example, the binding site of ERA (PDB code 1gwr) was obtained from the estradiol (Fig. 1C). *i*GEMDOCK is able to automatically consider the effects of hydrogen atoms when preparing the binding site and the compound library. In addition, *i*GEMDOCK allows users to visualize and refine the binding site of the target protein. Additionally, *i*GEMDOCK offers the similar way to prepare screening compounds and docking parameters (*e.g.*, the population size and the number of generations).

After the screening process, *i*GEMDOCK utilizes the post-screening analysis module to infer pharmacological interactions and cluster screening compounds based on protein-ligand complexes and compound structures (Fig. 1D). First, *i*GEMDOCK generates interaction profiles and calculates the pharmacological preference (*W_j_*) of each interacting group for deriving the pharmacological interactions (Fig. 2). These pharmacological preferences and interactions are shown in an interactive window (Fig. 2G); furthermore, RasMol displays the pharmacological interactions with conserved interacting residues and functional groups of compounds (Fig. 2H). Additionally, *i*GEMDOCK supports a hierarchical clustering method to cluster screening compounds according to interaction profiles and the atomic composition (Fig. 1E). The atomic composition, which is similar to the amino acid composition of a protein sequence, is useful for measuring compound similarity. *i*GEMDOCK provides an interactive interface for visualizing compound similarity with a hierarchical tree by Java Treeview. Finally, *i*GEMDOCK ranks and visualizes the screening compounds by combining the pharmacological interactions and the energy-based scoring function.

## Results and discussion

### Pharmacological interactions

**Figure 3:**
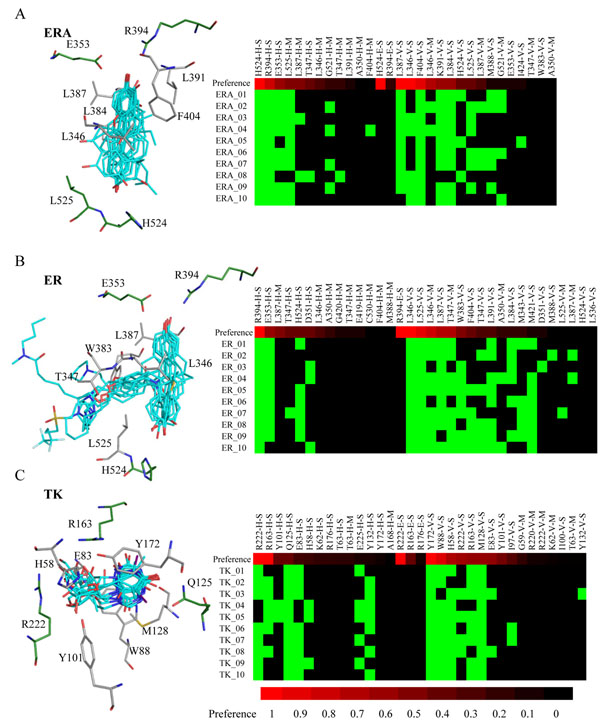
**Relationship between the pharmacological interactions and the active compounds of (A) ERA, (B) ER, and (C) TK.** The residue with a pharmacological preference ≥ 0.4 is colored by the interaction types [H: green (E353 and R394 in ERA); E: yellow; and V: gray (L391 and F404 in ERA)]. In the profile, the first row presents the pharmacological preferences of the interacting residue groups using the color-coding bar, with red-through-black indicating high-through-low. The following rows show the interactions between the active compounds and the interacting residue groups. H, E, and V indicate the interaction types; M and S indicate the main chain and the side chain of the interacting residue, respectively. The hydrogen-bonding or electrostatic interactions are colored in green if the energy ≤ -2.5. The vdW interactions are colored in green when the energy is less than -4.

The pharmacological interactions derived by *i*GEMDOCK are often involved in biological reactions or essential for ligand binding. We examined the pharmacological interactions on three selected target proteins (ERA, ER, and TK). First, we compared the pharmacological interactions, derived from 1000 screening compounds, to the consensus interactions, derived from 10 active compounds (Table 1 and Fig. 3). Here, the residue *i* is considered as "hot spot" if the consensus interaction ratio ≥0.5 [9,10,24,25]. Among 10 predicted pharmacological interactions (residues) for ERA, 9 pharmacological interactions (9 of 9 residues) agree with hot spots except the L387 with the hydrogen-bonding interaction. For TK, 8 of 14 pharmacological interactions (7 of 9 residues) are the hot spots. These results indicate the pharmacological interactions (residues) from screening compounds are often essential for the ligand binding. For example, 10 active compounds of TK form stacking interactions with the residue Y172 (vdW preference is 1.0 defined in Equation (1)) that stabilizes the binding of thymine or purine moieties.

**Table 1:** Pharmacological interactions and consensus interaction ratio on estrogen receptor α and thymidine kinase

Protein	Predicted pharmacological interactions	Consensus interaction ratio ^a^	Related works
ERA	R394-H^b^ (0.80^c^)	1.0	Form hydrogen bonding networks for ligand binding [26,27]
	E353-H (0.64)	0.8	
	H524-H (1.00)	1.0	
	
	L387-V (1.00)	0.9	Form non-polar contacts with A-ring of sterols scaffolds [28,29].
	L387-H (0.52)	0.2	
	F404-V (0.90)	1.0	
	
	V346-V (0.98)	0.5
	
	L391-V (0.61)	0.9
	L384-V (0.57)	0.9
	L525-H (0.53)	0.9

TK	R222-H (1.00)	0.8	Transfer phosphate in the substrate phosphorylation [30,31,36]
	R222-E (1.00)	0.0	
	R222-V (0.62)	0.4	
	R163-H (0.99)	0.6	
	R163-E (0.40)	0.0	
	R163-V (0.56)	0.8	
	E83-V (0.54)	0.2	
	
	Y101-H (0.40)	0.2	Form hydrogen bonds with thymidine; activity was decreased over 90% if Q125 mutated [32]
	Y101-V (0.45)	0.0	
	Q125-H (0.40)	1.0	
	
	Y172-V (1.00)	1.0	Sandwich the thymine moiety of substrates [33]
	M128-V (0.58)	1.0	
	
	W88-V (0.87)	0.9	Constitute a pocket for ligand binding [33]
	H58-V (0.68)	0.9	

^a^ The consensus interaction ratio of the residue *i* is defined as *A_j_*/*A*, where *A_j_* is the number of active compounds interacting to the residue *i* and *A* is total number of active compounds.

^b^ H, E and V are the interaction types.

^c^ The pharmacological preferences (i.e. *W_j_* defined in Equation (1)).

We also examined the pharmacological interactions by their biological functions or binding mechanisms. For estrogen receptor α, H524 (hydrogen-bonding preferences are 1.0 and 0.42 for ERA and ER, respectively) is involved in a hydrogen-bonding network [26]; similarly, E353 and R394 (hydrogen-bonding preferences ≥ 0.5 for both ERA and ER) interact the structural water to form the hydrogen bonding network (Table 1 and Fig. 3) [27]. These two hydrogen bonding networks are essential for estrogen receptor modulators to trigger the responses of estrogen receptor α[26,27]. For ER and ERA, hydrophobic interacting residues, L346, L387, F404, and L525 with high vdW interaction preferences, contact with the sterols or flavones scaffolds of the active compounds. These residues contribute the major vdW interactions for the ligand binding of estrogen receptor α [28,29].

For TK, R222 and R163 play major roles for inhibitor and substrate binding [30,31], and their hydrogen-bonding preferences are 1.0 and 0.99, respectively (Table 1). Our method identified the electrostatic interactions of R222 and R163 (preferences are 1.0 and 0.4, respectively), which help to transfer phosphate in the substrate phosphorylation [30]. However, these two electrostatic interactions are not observed from 10 active compounds (Fig. 3). For the residue Q125 (H preference 0.40), the TK activity was decreased over 90% if Q125 mutated into Asp, Glu, or Asn [32]. The residues M128, Y172, H58, R163, and Y88 constitute a pocket to fix the substrate, and their vdW preferences are 0.58, 1.00, 0.68, 0.56, and 0.87, respectively (Table 1). For the substrate binding, M128 and Y172 sandwich the thymine moiety and W88 is a part of the quasi-helical motif [33,34]. These results demonstrated that the pharmacological interactions derived by *i*GEMDOCK are often involved in the biological functions and the ligand binding.

### Molecular docking and virtual screening

To initially evaluate the utility of *i*GEMDOCK for docking and virtual screening, we selected a highly diverse dataset with 305 protein-ligand complexes (*i.e.*, CCDC/Astex set [20] ) and ERA, ER, and TK with 1000 compounds as test sets. Please note that the docking and screening tool of *i*GEMDOCK is GEMDOCK which was well-studied for VS and some applications [4,8,11-14]. In order to compare with previous works, we followed the docking procedures and performance indices proposed by Nissink, *et al*. A docked result was considered as a success solution if the root-mean-square derivation (RMSD) ≤2.0 Å between the docked solutions and X-ray crystal structures. For these 305 complexes, the success rates of *i*GEMDOCK and GOLD are 78% and 68%, respectively (Table S1 in additional file 1).

The pharmacological scoring function was then applied to identify the active compounds from the 1000 compounds of ERA, ER, and TK. Furthermore, we compared the screening results with those of using the energy-based scoring function of GEMDOCK. These two approaches were tested on the same datasets. The true hits of the three testing sets were used to access the screening accuracy of the two approaches (Fig. S1 in additional file 1). The screening accuracy was generally improved when *i*GEMDOCK considered the pharmacological interactions.

We compared *i*GEMDOCK (pharmacological scoring function) with three screening methods (DOCK, GOLD, and FlexX) on the ER and TK sets (Table S2 in additional file 1). To compare with previous works, we followed the experiment design and performance indices used by Bissantz *et al.* When true-positive rate is 80%, the false positive rates were 2.3% (*i*GEMDOCK), 13.3% (DOCK), 57.8% (FlexX), and 5.3% (GOLD), for ER. The false positive rates were 7.8% (*i*GEMDOCK), 23.4% (DOCK), 8.8% (FlexX), and 8.3% (GOLD) for TK.

### Post-screening analysis

To identify leads from vast amount of docked poses generated during the virtual screening procedure is the key step for the drug discovery. In addition, the top-ranked compounds based on the scoring functions are not advisable since these compounds may be similar in structures or physico-chemical properties. For these two issues, *i*GEMDOCK provides a post-screening analysis module to cluster compounds based on the interactions profiles and the atomic compositions. Selecting representative compounds from each cluster is able to maintain compound diversity and then reduces the false positives. Further, when active compounds are available, users can choose the similar compounds in the same cluster of the actives based on hierarchical trees (Fig. 1E). Notably, *i*GEMDOCK visualizes the interaction profiles of the compounds, and thereby the top-ranked compounds with pharmacological interactions can be selected according to the interaction table (Fig. 2G).

**Figure 4:**
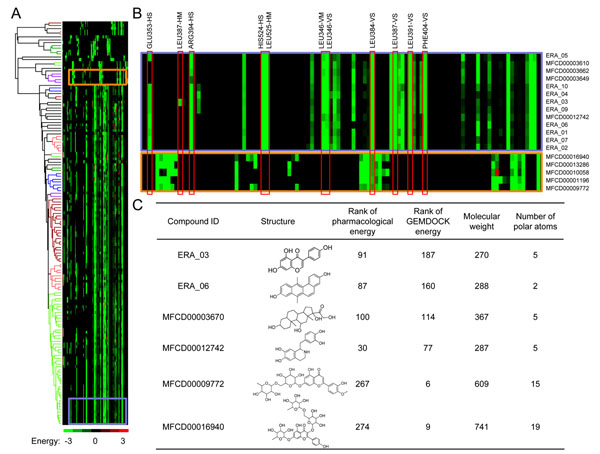
**Hierarchical clustering of iGEMDOCK for post screening analysis using ERA as the example.** (A) Hierarchical clustering of the interaction profile for the active compounds and top-ranked compounds selected by the pharmacological and energy-based scoring functions. (B) The interaction profiles of the compounds belonging to the active compound cluster (blue block) and the compounds in the lowest energy cluster (orange block). The red boxes present the pharmacological interactions. (C) The ranks of some typical compounds in the previous two clusters.

The post-screening analysis module of *i*GEMDOCK is useful for clustering and selecting compounds based on interaction profiles. We selected a set of compounds, including 10 ERA active compounds and top-ranked 100 compounds based on both the pharmacological and energy-based scoring functions. The hierarchical clustering dendrogram and the profile revealed that the protein-ligand interactions derived from the pharmacological scoring function are significantly different from those derived from the energy-based scoring function (Figs. 4A and B). The compounds with the high pharmacological scores and the active compounds consistently keep the pharmacological interactions; conversely, the compounds derived from the energy-based scoring function are often lack of the pharmacological interactions (Fig. 4B). This result indicates the pharmacological interactions are useful for identifying active compounds. For example, the two active compounds, ERA_03 and ERA_06, were ranked as 187 and 160 using the energy-based scoring function, respectively. When the pharmacological interactions were considered, the ranks of ERA_03 and ERA_06 were 91 and 87, respectively (Fig. 4C).

**Figure 5:**
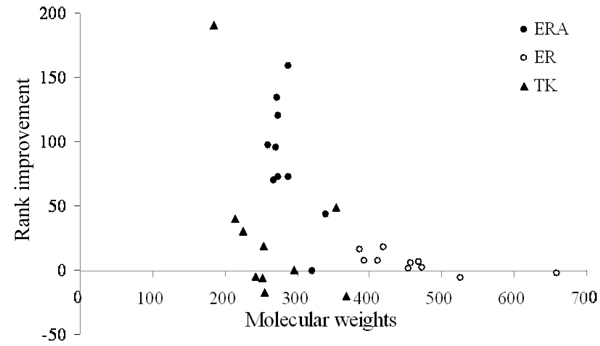
Relationship between the molecular weights of the active compounds and the rank improvement using the pharmacological scoring function for ERA (●), ER (○), and TK (▲).

Some compounds having high pharmacological scores are structurally and chemically similar to the active compounds (Fig. 4C). For example, MFCD00003670 (Tetrahydrocortisol) and MFCD00012742 (Tetrahydropapaveroline) were analogues of the ERA active compounds, and both of them were clustered into the same cluster. In addition, the pharmacological scoring function can reduce the ill-effect of most energy-based scoring functions which often favor high molecular weight or highly-polar compounds [8,35]. For instance, the ranks of two high molecular weight and polar compounds, MFCD00009772 and MFCD00016940, are 267 and 274, respectively (Fig. 4C). To further examine the pharmacological scoring function, we analyzed the relationship between the molecular weights of the active compounds and the rank improvement using the pharmacological scoring function (Fig. 5). The pharmacological scoring function generally improves the screening accuracy when the molecular weights of the active compounds are less than 400.

In summary, *i*GEMDOCK can mine the pharmacological interactions from the screening compounds without known active compounds. Therefore, *i*GEMDOCK can provide a good starting point for deriving pharmacological interactions (residues) and identifying new potential active compounds for a new protein structure. In addition, *i*GEMDOCK offers the visualization of the interaction profiles, pharmacological interactions, and the hierarchical clustering dendrogram. Users are able to easily observe and select compounds for post-screening analysis to enrich accuracies.

## Conclusions

We have developed a structure-based VS framework *i*GEMDOCK from the preparations through to the post-screening analysis. *i*GEMDOCK is an integrated and easy-to-use environment which is especially useful for post-screening analysis and inferring pharmacological interactions from screening compounds. The friendly user interface is helpful to biologically oriented nonexperts. The experimental results show that the pharmacological interactions are often essential for the binding of the active compounds and involved in biological mechanisms. The pharmacological interactions can reduce the ill effects of energy-based scoring functions to enrich the hit rates in VS. We believe *i*GEMDOCK is useful for drug discovery and understanding protein-ligand mechanisms.

## Competing interests

The authors declare that they have no competing interests.

## Authors' contributions

KCH, YFC, and JMY conceived and designed the experiments. YFC, SRL, and JMY implemented the program. KCH, YFC, and JMY performed the experiments and analyzed the data. KCH, YFC, and JMY wrote the paper.

## Supplementary Material

Additional file 1 Click here for file
