# Supplementary material for: iGEMDOCK: a graphical environment of enhancing GEMDOCK using pharmacological interactions and post-screening analysis
Source: BMC Bioinformatics. 2011 Feb 15;12(Suppl 1):S33. doi: 10.1186/1471-2105-12-S1-S33 (PMC3044289; doi:10.1186/1471-2105-12-S1-S33)
Supplement: Additional file 1 [file 1471-2105-12-S1-S33-S1.pdf]

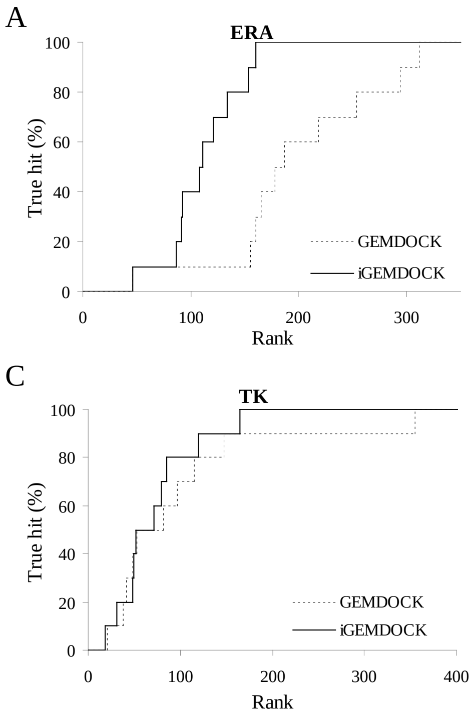

Supplementary Figure S1 - Comparison of iGEMDOCK (pharmacological scoring function) with GEMDOCK (energy-based scoring function) for (A) ERA, (B) ER, and (C) TK.

Supplementary Table S1 - Comparing iGEMDOCK with GOLD on the CCDC/Astex set by using the success rate

	*i*GEMDOCK				GOLD[a]			
RMSD	All entries[b]	Clean set [c]	Clean set with R<2.5Å[c]	Clean set with R<2.0 Å[c]	All entries[b]	Clean set [c]	Clean set with R<2.5Å[c]	Clean set with R<2.0 Å[c]
<0.5 Å	14 %	16 %	17 %	22 %	14 %	17 %	19 %	19 %
<1.0 Å	51 %	55 %	55 %	57 %	44 %	50 %	51 %	56 %
<1.5 Å	71 %	73 %	73 %	75 %	59 %	65 %	66 %	72 %
<2.0 Å	78 %	82 %	83 %	83 %	68 %	72 %	73 %	78 %
<2.5 Å	84 %	86 %	86 %	87 %	75 %	78 %	80 %	85 %
<3.0 Å	86 %	89 %	89 %	89 %	80 %	82 %	83 %	88 %

[a] Summarized from Nissink *et al* (Nissink, *et al*., 2002).
[b] 305 complexes.
[c] The clean set contains three subsets: all clean complexes (224 complexes), the complexes (180 complexes) with resolution (R) < 2.5 Å, and the complexes (92 complexes) with resolution < 2.0 Å.

Supplementary Table S2 - Comparing the pharmacological scoring function with other methods on ER and TK by the false-positive rates (%)

Protein	TP (%)[a]	*i*GEMDOCK[b]	GEMDOCK	DOCK[c]	FlexX[c]	GOLD[c]
ER	80	2.3[d]	3.1	13.3	57.8	5.3
	90	3.5	3.9	17.4	70.9	8.3
	100	5.2	7.0	18.9	NA	23.4
TK	80	7.8	10.8	23.4	8.8	8.3
	90	11.2	14.0	25.5	13.3	9.1
	100	15.7	34.9	27.0	19.4	9.3

[a] The true-positive rate (percentage).
[b] The pharmacological scoring function.
[c] Summarized from Bissantz *et al*.
[d] The false-positive rate from 990 random compounds (percentage).
